# Hypertension Control and Guideline-Recommended Target Blood Pressure Goal Achievement at an Early Stage of Hypertension in the UAE

**DOI:** 10.3390/jcm11010047

**Published:** 2021-12-23

**Authors:** Akshaya Srikanth Bhagavathula, Syed Mahboob Shah, Abubaker Suliman, Abderrahim Oulhaj, Elhadi Husein Aburawi

**Affiliations:** 1 Institute of Public Health, College of Medicine and Health Sciences, UAE University, Al Ain P.O. Box 17666, Abu Dhabi, United Arab Emirates; syeds@uaeu.ac.ae (S.M.S.); ababaker.suliman@uaeu.ac.ae (A.S.); aoulhaj@uaeu.ac.ae (A.O.); 2 Department of Pediatrics, College of Medicine and Health Sciences, UAE University, Al Ain P.O. Box 17666, Abu Dhabi, United Arab Emirates

**Keywords:** blood pressure, hypertension, guidelines, targets, goals, cardiovascular, incident hypertension, United Arab Emirates, Middle East, Arabian Gulf

## Abstract

(1) Background: The present study aimed to assess the changes in blood pressure (BP) within the first 6 months of treatment initiation in a newly treated hypertensive cohort and to identify the factors that are associated with achieving the target BP recommended by the American (ACC/AHA, 2017), European (ESC/ESH, 2018), United Kingdom (NICE, 2019), and International Society of Hypertension (ISH, 2020) guidelines. (2) Methods: We analyzed 5308 incident hypertensive outpatients across Abu Dhabi, United Arab Emirates (UAE), in 2017; each patient was followed up for 6 months. Hypertension was defined as a BP of 130/80 mmHg according to the ACC/AHA guidelines and 140/90 mmHg according to the ESC/ESH, NICE, and ISH guidelines. Multiple logistic regression was used to identify factors associated with achieving the guideline-recommended BP targets. (3) Results: At baseline, the mean BP was 133.9 ± 72.9 mmHg and 132.7 ± 72.5 mmHg at 6 months. The guideline-recommended BP targets were 39.5%, 43%, 65.6%, and 40.8%, according to the ACC/AHA, ESC/ESH, NICE, and ISH guidelines, respectively. A BMI of <25 kg/m^2^ was associated with better BP control according to the ACC/AHA (odds ratio (OR) = 1.26; 95% confidence interval (CI) = 1.07–1.49), ESC/ESH (OR = 1.27; 95% CI = 1.08–1.50), and ISH guidelines (OR = 1.22; 95% CI = 1.03–1.44). Hypertension treated in secondary care settings was more likely to achieve the BP targets recommended by the ACC/AHA (1.31 times), ESC/ESH (1.32 times), NICE (1.41 times), and ISH (1.34 times) guidelines. (4) Conclusions: BP goal achievement was suboptimal. BP control efforts should prioritize improving cardiometabolic goals and lifestyle modifications.

## 1. Introduction

High blood pressure (BP) or hypertension is a leading risk factor for cardiovascular (CV) morbidity and mortality [1,2]. It is estimated that over 10 million deaths occur every year due to high BP [1]. Maintaining an optimal BP (120/80 mmHg) can help reduce the risk of CV, cerebrovascular, and renal complications significantly [3,4,5,6,7,8,9,10,11]. Numerous clinical trials and meta-analyses have demonstrated that maintaining a systolic BP (SBP) of 120 mmHg and a diastolic BP (DBP) of 80 mmHg has several benefits for reducing the risk associated with cardiovascular disease (CVD) [4,10,12,13,14,15]. Therefore, clinical guidelines have evolved to recommend lower BP thresholds, combined with lifestyle changes and antihypertensive drug therapy [16,17,18,19]. In addition, hypertension management guidelines have proposed the control of some cardiometabolic risk factors, such as lipids and glucose profile, and treatment targets for the associated CVD risk factors [16,17,18,19].

In the last 4 years, four essential BP guidelines have been published, including the American College of Cardiology/American Heart Association (ACC/AHA, 2017) BP guidelines [16], the European Society of Cardiology/European Society of Hypertension (ESC/ESH, 2018) guidelines [17], the National Institute for Health and Care Excellence’s hypertension in adults guidelines (NICE, 2019) [18], and the most recent International Society of Hypertension (ISH, 2020) Global Hypertension Practice Guidelines [19]. Each set of guidelines aims to improve the diagnosis and control of hypertension by redefining the optimal systolic/diastolic BP targets for treatment in line with the most recent evidence.

With a population of 9.2 million in the United Arab Emirates (UAE), the age-standardized prevalence of hypertension was 33% in 2019, and less than half (47%) of patients were on treatment, and the control group comprised only 19% [20,21,22,23]. However, most of the practitioners in the UAE are expatriates and apply various clinical guidelines in their clinical practices. The differences in international guidelines may potentially influence the diagnosis and treatment targets worldwide, including in Arabian countries, where most physicians follow various international guidelines. Moreover, no data are available to investigate the achievement of BP goals in newly diagnosed hypertensive patients, according to the international guidelines. To the best of our knowledge, no study has been conducted to assess the achievement of the guideline-recommended BP goals among incident hypertensive patients in the Middle East. The current study aimed to assess the changes in BP after 6 months of antihypertensive treatment, starting from initiation, and compare the achievement of BP treatment targets with the recommendations of each of the guidelines cited above. It also aimed to identify the factors associated with achieving BP goals according to each guideline.

## 2. Materials and Methods

The study cohort, study population, inclusion and exclusion criteria, and baseline data including anthropometric and BP measurements were previously described in detail [24]. Briefly, a retrospective chart review was conducted to identify incident hypertensive adults registered for antihypertensive treatment across Abu Dhabi Health Services (SEHA) between 1 January 2017 and 31 December 2017, and each patient was retrospectively followed up for 6 months to assess their outcomes. The chart review was conducted from 1 September 2018 until 31 October 2019. Patients with incident hypertension who were diagnosed by a consulting physician using 24-h ambulatory BP monitoring or home-based BP measurements and who subsequently started antihypertensive treatment were considered as the target population.

All the SEHA facilities are equipped with automated unattended BP devices that give an average of several measurements. According to the SEHA protocol, each patient rests in a sitting position for 5 min in a separate room, and the measurement procedure yields three consecutive measurements. The mean of these three measurements obtained at baseline and at the 6-month follow-up examination was used in this study. Moreover, the physician should use the automated office BP measurements to initiate therapy or repeat the prescription for medication. Patients with prior use of antihypertensive medication or hospitalization due to elevated BP in the past 12 months of the index period were excluded.

### 2.1. Operational Definitions

Hypertension is defined as an SBP/DBP of at least 130/80 mmHg according to ACC/AHA guidelines and 140/90 mmHg according to the ESC/ESH, NICE, and ISH guidelines, with current use of antihypertensive medications. The classifications of hypertension and the recommended BP targets according to the international guidelines are illustrated in Table 1.

### 2.2. Data Variables

Along with the BP parameters, the data collected at baseline and at 6-month follow-up included age, sex, healthcare center location (rural/urban), type of health facility (primary, secondary, or tertiary), smoking status (smoker/non-smoker), body mass index (BMI, kg/m^2^), and diabetes status.

### 2.3. Statistical Analysis

The baseline characteristics were presented as frequencies and percentages for categorical variables. Means plus/minus standard deviations (SD) were used for continuous variables. The chi-square test was used to compare the differences between the groups for categorical variables, and ANOVA or the Kruskal-Wallis test was used for continuous variables, as appropriate. Factors independently associated with changes in BP at 6-month follow-up were determined using Wilcoxon’s signed-rank test and the Mann-Whitney U test or Kruskal-Wallis test, as appropriate. Univariate and multivariate logistic regression analyses were performed to identify the factors associated with increasing the likelihood of achieving BP targets according to each guideline. Odds ratios (OR) with 95% CIs were calculated. A *p*-value of <0.05 was considered statistically significant, and all tests were two-sided. The analysis was performed using SPSS package 24.0 (IBM Corp., Armonk, NY, USA).

## 3. Results

In total, 5308 patients aged 18 years or above who registered for hypertension treatment across 54 SEHA facilities in Abu Dhabi, UAE, were identified. The baseline characteristics of these patients are summarized in Table 2. Subjects had a mean age of 54.8 ± 11.5 years and a BMI of 31.2 ± 7.5 kg/m^2^. The majority of patients were women (53.7%) and received care in primary care centers (60.1%). A higher baseline BP (≥140/≥90 mmHg) was significantly associated with higher BMI and treatment in primary care settings.

**Figure 1:**
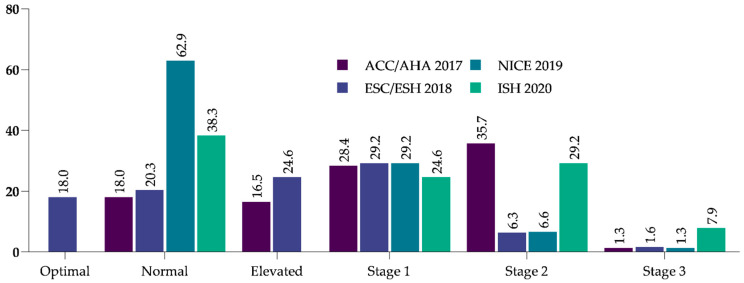
Distribution of blood pressure according to international guidelines at baseline. ACC/AHA: American College of Cardiology/American Heart Association; ESC: European Society of Cardiology/European Society of Hypertension; NICE: National Institute of Health Care and Excellence (UK); ISH: International Society of Hypertension.

At baseline, 28.4%, 29.2%, 29.2%, and 24.6% of the subjects had stage 1 hypertension according to the ACC/AHA, ESC/ESH, NICE, and ISH guidelines, respectively. More details on the distribution of BP according to international guidelines are shown in Figure 1.

**Figure 2:**
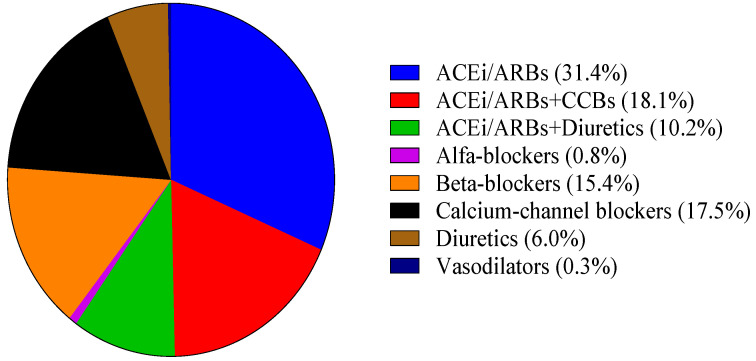
Antihypertensive drug classes prescribed.

Among those newly diagnosed as hypertensive, different classes of antihypertensive drugs were prescribed as monotherapy (71.5%) or combination therapy (28.4%). The most frequently used antihypertensive drugs were angiotensin-convertase inhibitors/angiotensin receptor blockers (ACEi/ARBs) (31.4%), followed by calcium channel blockers (CCBs) (17.5%) and beta-blockers (15.4%). Furthermore, the proportion prescribed a combination of ACEi/ARBs with CCBs or diuretics was 18.1% and 10.2%, respectively. More details are given in Figure 2.

### 3.1. Changes in the BP

At 6-month follow-up, a significant average reduction in SBP of −1.68 mmHg (95% CI: −2.46 to −0.91; *p* < 0.001), and a reduction in DBP of −1.15 mmHg (95% CI: −1.69 to −0.60; *p* < 0.001) was observed in men. For women, a significant reduction was observed in SBP by −0.84 mmHg (95% CI: −1.57 to −0.12, *p* = 0.046) but not in DBP (0.22 mmHg) (95% CI: −0.29 to 0.73, *p* = 0.795). The average changes in DBP and SBP were significantly different between men and women (*p* < 0.001, *p* = 0.035, respectively). Moreover, significant differences in SBP (*p* < 0.001) and DBP (*p* < 0.001) were observed across healthcare settings. The hypertensive patients treated in primary care settings showed a significant reduction in SBP by −2.31 mmHg, (95% CI: −2.99 to −1.63; *p* < 0.001) and in DBP by −2.15, (95% CI: −2.60 to −1.70; *p* < 0.001); however, no such differences in SBP were observed for patients treated in secondary and tertiary care settings, as shown in Table 3. Moreover, no significant differences in SBP and DBP were observed between the groups in terms of healthcare center location, BMI, and smoking status.

### 3.2. Achievement of BP Goals According to International Guidelines

**Figure 3:**
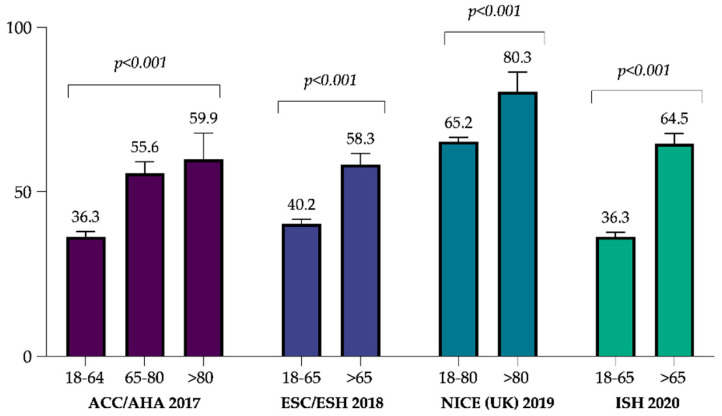
Proportion of hypertensive patients achieving the target blood pressure, stratified by age, at 6-month follow-up according to the different international guidelines. ACC/AHA: American College of Cardiology/American Heart Association; ESC: European Society of Cardiology/European Society of Hypertension; NICE: National Institute of Health Care and Excellence (UK); ISH: International Society of Hypertension.

The overall BP goal achievements by the 6-month follow-up examination was 39.5% (95% CI: 38.2–40.9), according to the ACC/AHA guidelines, 43% (95% CI: 41.6–44.3) for the ESC/ESH guidelines, 65.7% (95% CI: 64.4–66.9) for the NICE guidelines, and 40.9% (95% CI: 39.5–42.3) for the ISH guidelines. Figure 3 summarizes the proportion of patients, stratified by age, who achieved the BP goals recommended by each set of guidelines. According to the ACC/AHA guidelines, only 36.3% (95% CI: 34.9–37.7) of the patients aged 18–64 years reached the BP goal of <130/80 mmHg and 56.3% (95% CI: 52.9–59.6) of patients aged 65 years or older achieved the BP goal of <140/80 mmHg.

The achievement of BP goals according to each set of guidelines was stratified by age, smoking status, BMI, and diabetes status, as presented in Table 4. Only 31.7% of patients aged ≤40 years achieved the ACC/AHA-recommended BP goal; moreover, 45.4% of smokers and 37.5% of those with a BMI of ≥25 kg/m^2^ achieved the ACC/AHA-recommended BP goal (<130/80 mmHg).

### 3.3. Factors Associated with the Achievement of Guideline-Recommended BP Goals

The factors that are significantly associated with achievement of each guideline-recommended BP goal in hypertensive patients are shown in Table 5. The unadjusted logistic regression analysis indicated that a normal BMI of <25 kg/m^2^ is a significant predictor of achieveing the ACC/AHA-recommended BP goals (OR: 1.32, 95% CI: 1.12–1.55). Similar findings were observed for the ESC/ESH (OR: 1.31; 95% CI: 1.11–1.54) and ISH (OR: 1.29; 95% CI: 1.09–1.52) goals. On the other hand, the ESC/ESH and NICE goals were associated with secondary care settings (OR: 1.23; 95% CI: 1.03–1.47, and OR: 1.26; 95% CI: 1.04–1.52).

After adjusting for multiple covariates, the guideline-recommended BP goal was more likely to be achieved by those who maintained a normal BMI and were treated in secondary healthcare settings. Briefly, those with a BMI of <25 kg/m^2^ were associated with achievement of the BP targets recommended by the ACC/AHA guidelines (OR: 1.26, 95% CI: 1.07–1.49), the ESC/ESH guinelines (OR: 1.27, 95% CI: 1.08–1.50), and the ISH guidelines (OR: 1.22, 95% CI: 1.03–1.44). More details are shown in Table 5.

## 4. Discussion

This is the first study to report contemporary data regarding changes in BP and the achievement of guideline-recommended targets for BP in newly diagnosed hypertensive patients. It also identified factors that could increase the likelihood of achieving the BP targets according to each guideline. After 6 months of treatment, the overall average reduction in SBP and DBP was only 1.23 mmHg and 0.4 mmHg, respectively. Moreover, the current study found that 60% of the subjects did not achieve the target BP recommended by the ACC/AHA guidelines, in contrast to subjects meeting the ESC/ESH guidelines (43%), the NICE guidelines (65.7%), and the ISH guidelines (40.9%). Notably, achievement of the guideline-recommended BP targets among young patients (≤40 years), smokers, those with a BMI of ≥25 kg/m^2^ and ≥30 kg/m^2^, and people with diabetes was dramatically low. Those who had a normal BMI and were treated in secondary care settings were more likely to achieve the guideline-recommended BP targets.

The primary analysis of this study focused on changes in BP after 6 months of antihypertensive treatment, starting from initiation. The results showed a broad difference in changes in SBP (−1.23 mmHg) and DBP (−0.4 mmHg) in 6 months. The magnitude of the BP changes in 6 months varied across the study population on the basis of age, sex, and healthcare setting. To the best of our knowledge, no studies in the UAE or the Middle East have used longitudinal data to date to investigate the changes in BP in the Arabian population. Prior studies from the Western countries demonstrated that greater visit-to-visit changes in BP over time were associated with an increased risk of CVD events, irrespective of the mean BP level [25,26]. A recent SPRINT ancillary study indicated the potential for more significant differences in the mean SBP between trial participants by −7.3 mmHg (95% CI: −7.6 mmHg to −7.1 mmHg) and those receiving standard care (−4.6 mmHg, 95% CI: −4.9 mmHg to −4.4 mmHg) after 1 year [27]. In addition, the differences between the routine BPs and trial BPs were not consistent over time and also varied significantly by sex, history of CVD, and clinical site. This highlights the differences in BP measurement techniques, the quality of estimates, and clinical practices between trials and routine clinical settings. However, most of the SPRINT trial participants were older adults (mean age: 68.5 ± 9.1 years) and had a history of CVD [27,28]. This limits our ability to generalize or translate these findings to the present population, who are at the early stage of hypertension, with a mean age of 54.8 ± 11.5 years.

Adopting a lower BP threshold for hypertension (≥130/80 mmHg) and setting stringent target BP goals emphasizes the importance of early preventive measures and non-pharmacological interventions. Despite this, studies continue to record inadequate control of BP [20,23,24,28,29,30,31] and show poor physician compliance with such guidelines [32,33,34]. The continental divide of guideline-recommended BP goals is reflected in our study and showed variations in achievement across hypertensive subjects. When we applied the ACC/AHA criteria (<130/80 mmHg) to our population, there was a substantially lower proportion of patients (39.5%) who achieved a consistent BP target compared with the rates reported in the USA (46.6%) [35], Canada (41.1%) [36], and Spain (25.1%) [37]. However, when using the BP targets of the ESC/ESH, NICE, and ISH criteria, the achievement of BP goals was similar or lower than that in Italy (66.5%) [38] and the UK (63%) [39] but higher than the 2010 level of global BP control (13.8%) [1].

The current study data revealed that achievement of the BP goals recommended by the four different guidelines across hypertensive patients in different age groups was substantially low. Furthermore, a marginal difference in the achievement of guideline-recommended BP goals was observed between the groups. These variations were also reflected across all the guidelines compared. Although the underlying reasons for the association between the groups and less improved BP control were unclear, this putative relationship warrants further elucidation. The improved BP control among these patients may be partly explained by the comprehensive promotion of a healthy lifestyle, such as adequate physical activity, low saturated fat intake, low-salt meals, and medical information provided by counselors regarding the CVD risks related to hypertension.

The notable findings of this study are the overall high rate of BP goal achievement among patients maintaining a normal BMI. These patients are more likely to achieve the guideline-recommended BP targets (except for the NICE recommendations). As shown in the guidelines, the first step of intervention in newly diagnosed hypertensive patients is lifestyle changes such as consuming a heart-healthy diet (DASH), increasing physical activity, and weight loss for those who are overweight or obese [16,17,18,19]. Our study demonstrated a positive relationship between optimal BMI and BP goal achievement. This indicates that as long as weight is still below the baseline level, there can be an impact on BP.

On the other hand, a recent Cochrane review observed that a 4.0-kg reduction in body weight reduced SBP and DBP by 4.5 mmHg and 3.2 mmHg, respectively, and significantly reduced cardiovascular mortality (HR: 0.70, 95% CI: 0.57–0.87) in hypertensive individuals [40]. A meta-analysis of 25 randomized controlled trials showed a reduction in BP of 1.05/0.92 mmHg on average for each kilogram of weight lost [41]. Regardless of whether BP values are above normal, a healthy diet and lifestyle modifications should be prioritized, along with adherence to pharmacological treatment, which could ultimately lower CVD risk in the early stage of hypertension.

In this study, about 15% of patients were managed in secondary care settings and were more likely to achieve the BP targets recommended by four different international guidelines. Additionally, the magnitude of the changes in BP in 6 months varied significantly for each setting. A study conducted by Billups et al. investigated the BP control among 86,512 hypertensive subjects in the USA by applying a BP cutoff of <140/90 mmHg [42]. They observed that patients treated in primary care settings were more likely to control their BP than those treated in specialty settings. The reason for these differences is not clear. In this regard, more research is needed to understand the determinants of BP control at the early stage of hypertension. The plausible reasons for higher BP control in the secondary care settings is that these are specialty clinic settings for chronic disease, and the physicians spend more time with patients explaining the importance of lifestyle modifications, medication adherence, and ultimately improved BP control.

### Strengths and Limitations

This is the first study that investigated hypertension control and achievement of BP goals in a nationally representative sample of hypertensive patients in the UAE since the 1998 NASH-UAE study [43]. Therefore, our findings may serve to reasonably compare the public health implications of each guideline in the UAE population.

The current study has some limitations. First, the present study relied on automated office BP measurements from an electronic medical record; however, this method of determining hypertension has been widely applied in previous studies [44,45,46], and office-based BP measurements are routinely used in the management of hypertension. Second, owing to the study’s retrospective nature, we were unable to obtain complete information regarding some of the factors associated with the achievement of guideline-recommended BP targets, including physical activity, some cardiometabolic risk factors, and diet to control hypertension. Third, we did not have data on patients with white-coat hypertension or masked hypertension. Fourth, although BP control was suboptimal, we did not investigate the potential causes of poor BP control. Fifth, it is hard to define the response to treatment within 6 months of treatment initiation, and more effort is needed to understand BP control in the incident hypertensive population. Finally, our data were collected from 2017–2018 across SEHA facilities in the emirate of Abu Dhabi, the largest emirate in the UAE, which may not represent the entire UAE.

## 5. Conclusions

This study showed suboptimal BP control across the study population and identified variations in the BP goals according to the ACC/AHA guidelines vs. the ESC/ESH, NICE, and ISH guidelines in the UAE population. Furthermore, the overall average reduction in BP within 6 months was only 1.23 (SBP) and 0.4 (DBP) mmHg. We found that hypertensive patients with a normal BMI and those who were treated in secondary care settings were more likely to achieve the guideline-recommended BP targets. BP control efforts should also prioritize improving cardiometabolic goals and lifestyle changes at an early stage of hypertension.

## Figures and Tables

**Table 1:** Classification of hypertension and BP targets according to the international guidelines.

	ACC/AHA 2017	ESC/ESH 2018	NICE 2019	ISH 2020
normal BP	systolic <120 mm Hg and diastolic <80 mm Hg	systolic 120–129 mm Hg and/or diastolic 80–84 mm Hg	<140 and/or <90 mm Hg	<130 mm Hg and/or diastolic <85 mm Hg
elevated BP	systolic 120–129 mm Hg and <80 mm Hg	systolic 130–139 and/or diastolic 85–89		high normal (systolic 130–139 mm Hg and/or diastolic 85–89 mm Hg)
stage 1 hypertension	systolic 130–139 mm Hg or diastolic 80–89 mm Hg	systolic 140–159 mm Hg and/or diastolic 90–99 mm Hg	systolic 140–159 mm Hg and/or diastolic 90–99 mm Hg	systolic 140/159 mm Hg and/or diastolic 90–99 mm Hg
stage 2 hypertension	systolic ≥ 140 mm Hg or diastolic ≥90 mm Hg	systolic 160–179 mm Hg and/or diastolic 100–109 mm Hg	systolic 160–179 mm Hg and/or diastolic 100–120 mm Hg	systolic ≥ 160 mm Hg and/or diastolic ≥ 100 mm Hg
hypertension crises	systolic >180 and/or diastolic >120	systolic ≥180 mm Hg and/or diastolic ≥110 mm Hg	systolic ≥180 mm Hg and/or diastolic ≥120 mm Hg	-
BP targets (age)				
18 to <65	<130/80 mmHg	130/80 mmHg (18–65 years)	<140/90 mmHg (18–80 years)	<130/80 mmHg
65–80	<140/80 mmHg	<140/80 mmHg (>65 years)		<140/90 mmHg
>80	<140/80 mmHg	-	<150/90 mmHg	-

BP: blood pressure; ACC/AHA: American College of Cardiology/American Heart Association; ESC/ESH: European Society of Cardiology/European Society of Hypertension; NICE: The National Institute for Health and Care Excellence; ISH: International Society of Hypertension.

**Table 2:** Characteristics of the study population according to the blood pressure levels at baseline.

Characteristics	Total	<120 and <80	120–129 and <80	130–139 or 80–89	≥140 or ≥90	*p*-Value
number (%)	5308 (100.0)	958 (18.0)	874 (16.5)	2099 (39.5)	1377 (25.9)	
age, mean (±SD)	54.8 (±11.5)	55.2 (±11.8)	55.3 (±11.9)	54.5 (±10.9)	54.8 (±12.1)	0.164
gender, *n* (%)						0.223
men	2459 (46.3)	422 (44.1)	391 (44.7)	996 (47.5)	650 (47.2)	
women	2849 (53.7)	536 (55.9)	483 (55.3)	1103 (52.5)	727 (52.8)	
health center location, *n* (%)						0.662
rural	2595 (48.9)	479 (50.0)	412 (47.1)	1027 (48.9)	677 (49.2)	
urban	2713 (51.1)	479 (50.0)	462 (52.9)	1072 (51.1)	700 (50.8)	
healthcare setting, *n* (%)						<0.001
primary	3189 (60.1)	468 (48.9)	488 (55.8)	1388 (66.1)	845 (61.4)	
secondary	795 (15.0)	220 (23.0)	135 (15.4)	243 (11.6)	197 (14.3)	
tertiary	1324 (24.9)	270 (28.2)	251 (28.7)	468 (22.3)	335 (24.3)	
smoking, *n* (%)						0.955
smoker	1883 (35.5)	346 (36.1)	313 (35.8)	740 (35.3)	484 (35.1)	
non-smoker	3425 (64.5)	612 (63.9)	561 (64.2)	1359 (64.7)	893 (64.9)	
BMI (kg/m^2^), mean (±SD)	31.2 (±7.5)	30.6 (±6.8)	30.6 (±6.3)	31.6 (±8.4)	31.4 (±6.9)	0.001
missing	720 (13.6)	159 (16.6)	116 (13.3)	236 (11.2)	209 (15.2)	
diabetes, *n* (%)						0.590
no	4437 (83.6)	796 (83.1)	730 (83.5)	1744 (83.1)	1167 (84.7)	
yes	871 (16.4)	162 (16.9)	144 (16.5)	355 (16.9)	210 (15.3)	

BMI: body mass index, SD: standard deviation.

**Table 3:** Changes in blood pressure at 6-month follow-up across the study subjects.

Characteristics	Change in SBP	*p*-Value *	*p*-Value **	Change in DBP	*p*-Value *	*p*-Value **
sex			0.035			<0.001
men	−1.68 (−2.46 to −0.91)	<0.001		−1.15 (−1.69 to −0.60)	<0.001	
women	−0.84 (−1.57 to −0.12)	0.046		0.22 (−0.29 to 0.73)	0.795	
age (years)			0.951			0.012
18–40	−0.81 (−2.48 to 0.85)	0.262		−0.72 (−1.96 to 0.52)	0.289	
41–50	−1.39 (−2.46 to −0.32)	0.013		−1.25 (−2.03 to −0.47)	0.002	
51–60	−1.03 (−1.83 to −0.22)	0.006		−0.09 (−0.64 to 0.45)	0.244	
61–75	−1.82 (−3.15 to −0.49)	0.016		−0.81 (−1.74 to 0.11)	0.104	
>75	−1.07 (−3.30 to 1.15)	0.246		2.17 (0.63 to 3.70)	0.027	
health center location			0.713			0.912
rural	−1.11 (−1.87 to −0.35)	0.004		−0.46 (−0.99 to 0.06)	0.039	
urban	−1.35 (−2.09 to −0.61)	<0.001		−0.37 (−0.90 to 0.16)	0.068	
healthcare setting			<0.001			<0.001
primary	−2.31 (−2.99 to −1.63)	<0.001		−2.15 (−2.60 to −1.70)	<0.001	
secondary	1.01 (−0.36 to 2.37)	0.144		3.38 (2.34 to 4.42)	<0.001	
tertiary	0.02 (−1.05 to 1.09)	0.877		1.48 (0.69 to 2.28)	<0.001	
BMI (kg/m^2^)			0.277			0.077
<18.5	−0.20 (−7.22 to 6.83)	0.909		0.78 (−3.46 to 5.02)	0.630	
18.5–25	−0.16 (−1.69 to 1.38)	0.646		0.22 (−0.89 to 1.32)	0.401	
25–30	−1.56 (−2.54 to −0.57)	<0.001		−0.74 (−1.42 to −0.05)	0.007	
>30	−0.89 (−1.65 to −0.13)	0.044		0.07 (−0.48 to 0.62)	0.939	
smoking			0.838			0.259
smoker	−1.32 (−2.21 to −0.42)	0.004		−0.75 (−1.37 to −0.14)	0.011	
non-smoker	−1.19 (−1.85 to −0.53)	<0.001		−0.23 (−0.70 to 0.24)	0.128	

* Wilcoxon signed-rank test, ** Mann-Whitney U test, or Kruskal-Wallis test.

**Table 4:** Percentage of adults who achieved the BP goals according to different international guidelines.

Characteristics		Total (%)	ACC/AHA 2017	ESC/ESH 2018	NICE 2019	ISH 2020
age (years)	≤40	536 (10.1)	31.7 (27.9–35.8)	37.1 (33.1–41.3)	61.6 (57.4–65.6)	31.7 (27.9–35.8)
	>40	4772 (89.9)	40.4 (39.0–41.8)	43.4 (42.0–44.8)	66.1 (64.8–67.4)	41.7 (40.3–43.1)
smoking	smoker	1883 (35.5)	45.4 (43.2–47.7)	47.4 (45.2–49.7)	66.7 (64.5–68.8)	48.6 (46.3–50.9)
	non-smoker	3245 (64.5)	36.3 (34.7–37.9)	40.2 (38.5–41.8)	65.1 (63.5–66.7)	36.3 (34.7–37.9)
BMI	<25 kg/m^2^	672 (12.6)	44.2 (40.5–48.0)	47.5 (43.7–51.2)	68.2 (64.5–71.6)	44.9 (41.2–48.7)
	≥25 kg/m^2^	3910 (73.6)	37.5 (36.0–39.1)	40.8 (39.3–42.4)	65.2 (63.7–66.7)	38.8 (37.3–40.3)
BMI	<30 kg/m^2^	2156 (47.1)	41.5 (39.4–43.6)	44.9 (42.9–47.1)	67.9 (65.9–69.8)	42.8 (40.7–44.9)
	≥30 kg/m^2^	2426 (52.9)	35.9 (34.0–37.8)	39.0 (37.1–41.0)	63.6 (61.7–65.5)	36.9 (35.0–38.9)
diabetes	no	4437 (83.6)	39.9 (38.5–41.4)	43.2 (41.8–44.7)	66.2 (64.8–67.6)	41.3 (39.8–42.7)
	yes	871 (16.4)	37.5 (34.4–40.8)	40.2 (37.0–43.5)	62.9 (59.7–66.1)	37.5 (34.4–40.8)

ACC/AHA: American College of Cardiology/American Heart Association; ESC/ESH: European Society of Cardiology/European Society of Hypertension; NICE: National Institute for Health and Care Excellence hypertension in adult guidelines; ISH: International Society of Hypertension; BMI: body mass index.

**Table 5:** Factors associated with the achievement of each guideline-recommended BP goals.

Variable	Odds Ratio (95% Confidence Interval)							
	ACC/AHA 2017		ESC/ESH 2018		NICE 2019		ISH 2020	
	Crude	Adjusted	Crude	Adjusted	Crude	Adjusted	Crude	Adjusted
age								
<65	0.44 (0.38–0.51)	0.47 (0.39–0.58)	0.54 (0.46–0.62)	0.59 (0.48–0.73)	0.92 (0.79–1.07)	1.04 (0.84–1.28)	0.33 (0.28–0.39)	0.34 (0.28–0.42)
≥65	1.00	1.00	1.00	1.00	1.00	1.00	1.00	1.00
sex								
men	1.06 (0.95–1.18)	0.99 (0.88–1.12)	1.04 (0.93–1.16)	0.97 (0.86–1.10)	1.00 (0.89–1.12)	0.95 (0.84–1.07)	1.06 (0.95–1.18)	1.00 (0.88–1.12)
women	1.00	1.00	1.00	1.00	1.00	1.00	1.00	1.00
health center location								
rural	1.02 (0.91–1.13)	0.93 (0.80–1.08)	1.04 (0.93–1.16)	0.96 (0.82–1.11)	1.07 (0.95–1.19)	0.97 (0.83–1.13)	1.01 (0.90–1.12)	0.92 (0.79–1.07)
urban	1.00	1.00	1.00	1.00	1.00	1.00	1.00	1.00
healthcare setting								
primary	0.96 (0.84–1.10)	1.09 (0.92–1.30)	0.99 (0.87–1.13)	1.12 (0.95–1.32)	1.05 (0.92–1.20)	1.15 (0.97–1.37)	0.96 (0.84–1.09)	1.11 (0.94–1.32)
secondary	1.19 (0.99–1.42)	1.31 (1.03–1.66) *	1.23 (1.03–1.47) *	1.32 (1.05–1.67) *	1.26 (1.04–1.52) *	1.41 (1.11–1.81) **	1.18 (0.99–1.42)	1.34 (1.05–1.70) *
tertiary	1.00	1.00	1.00	1.00	1.00	1.00	1.00	1.00
smoking status								
non-smoker	0.69 (0.61–0.77)	0.97 (0.83–1.13)	0.74 (0.66–0.83)	0.95 (0.82–1.11)	0.93 (0.83–1.05)	0.93 (0.80–1.09)	0.60 (0.54–0.68)	0.97 (0.83–1.13)
smoker	1.00	1.00	1.00	1.00	1.00	1.00	1.00	1.00
BMI (kg/m^2^)								
<25	1.32 (1.12–1.55) **	1.26 (1.07–1.49) **	1.31 (1.11–1.54) **	1.27 (1.08–1.50) **	1.14 (0.96–1.36)	1.14 (0.96–1.37)	1.29 (1.09–1.52) **	1.22 (1.03–1.44) *
≥25	1.00	1.00	1.00	1.00	1.00	1.00	1.00	1.00
diabetes								
yes	0.90 (0.78–1.05)	1.07 (0.90–1.26)	0.88 (0.76–1.02)	1.00 (0.85–1.17)	0.87 (0.75–1.01)	0.90 (0.76–1.07)	0.85 (0.74–0.99)	1.07 (0.90–1.26)
no	1.00	1.00	1.00	1.00	1.00	1.00	1.00	1.00

* *p* ≤ 0.05, ** *p* ≤ 0.01. Significant factors that increase the likelihood of achieving the guideline-recommended BP goals. ACC/AHA: American College of Cardiology/American Heart Association; ESC/ESH: European Society of Cardiology/European Society of Hypertension; NICE: National Institute for Health and Care Excellence’s Hypertension in Adults guidelines; ISH: International Society of Hypertension. Adjusted for age, type of health center, BMI, and smoking status.

## Data Availability

All the data is available from the corresponding author and can be obtained upon reasonable request.
